# Melanopsin-mediated pupil function is impaired in Parkinson’s disease

**DOI:** 10.1038/s41598-018-26078-0

**Published:** 2018-05-17

**Authors:** Daniel S. Joyce, Beatrix Feigl, Graham Kerr, Luisa Roeder, Andrew J. Zele

**Affiliations:** 1 Institute of Health and Biomedical Innovation, Queensland University of Technology (QUT), Brisbane, Australia; 2 Visual Science Laboratory, School of Optometry and Vision Science, Queensland University of Technology (QUT), Brisbane, Australia; 3 Medical Retina Laboratory, School of Biomedical Sciences, Queensland University of Technology (QUT), Brisbane, Australia; 4 Movement Neuroscience Program, Queensland University of Technology (QUT), Brisbane, Australia; 5 Queensland Eye Institute, Brisbane, Australia; 6 Department of Psychiatry and Behavioral Sciences, School of Medicine, Stanford University, Stanford, USA

## Abstract

Parkinson’s disease (PD) is characterised by non-motor symptoms including sleep and circadian disruption. Melanopsin-expressing intrinsically photosensitive Retinal Ganglion Cells (ipRGC) transmit light signals to brain areas controlling circadian rhythms and the pupil light reflex. To determine if non-motor symptoms observed in PD are linked to ipRGC dysfunction, we evaluated melanopsin and rod/cone contributions to the pupil response in medicated participants with PD (*n* = 17) and controls (*n* = 12). Autonomic tone was evaluated by measuring pupillary unrest in darkness. In the PD group, there is evidence for an attenuated post-illumination pupil response (PIPR) amplitude and reduced pupil constriction amplitude, and PIPR amplitudes did not correlate with measures of sleep quality, retinal nerve fibre layer thickness, disease severity, or medication dosage. Both groups exhibited similar pupillary unrest. We show that melanopsin- and the rod/cone-photoreceptor contributions to the pupil control pathway are impaired in people with early-stage PD who have no clinically observable ophthalmic abnormalities. Given that ipRGCs project to brain targets involved in arousal, sleep and circadian rhythms, ipRGC dysfunction may underpin some of the non-motor symptoms observed in PD.

## Introduction

In Parkinson’s disease (PD), non-motor symptoms can precede motor symptoms and include sleep disturbances and daytime sleepiness, fatigue, depressed mood and cognitive impairments^1,2^. The aetiology underlying sleep and circadian disturbances in PD is not well understood, but is hypothesised to include dysregulation of the circadian system due in part to reduced dopaminergic neurotransmission (for review see Videnovic and Golombek, 2013)^3^. In people with PD, a 4-fold reduction in melatonin expression has been observed without altered circadian phase^4^. In mouse models of the disease, suprachiasmatic nucleus (SCN) signalling is reduced. These studies suggest degradation of environmental light signal processing via the retinohypothalamic tract that projects from the retina to the SCN.

The origin of the retinohypothalamic tract is a novel class of photoreceptors in the eye called intrinsically photosensitive retinal ganglion cells (ipRGCs)^5–7^. IpRGCs account for less than 0.5% of all retinal ganglion cells^5,7^ yet project to over a dozen brain areas including those involved in circadian photoentrainment, sleep and mood regulation, the pupil light reflex^6,8–15^ and for image forming human vision^16^ including the perception of brightness^17,18^. The transmission of light signals to the brain by ipRGCs is initiated at two retinal sites, intrinsically via the endogenous melanopsin photopigment^8,12,19,20^ and extrinsically from rod and/or cone photoreceptors^6^ that involve dopaminergic amacrine intermediary cells^19,21–23^. Melanopsin has high sensitivity to short wavelength (blue) light with a physiological response characterised by slow temporal kinetics and sustained signalling after light cessation^6^; in humans, the kinetics of the pupil light reflex after stimulus offset (the post-illumination pupil response, PIPR) provide a signature, non-invasive measure of melanopsin function^24–27^ that can be differentiated from extrinsic photoreceptor inputs using non-invasive chromatic pupillometry^28–31^. Pupillometric assessment of ipRGCs in humans has shown clinical promise for a range of retinal and non-retinal diseases (for review see Feigl & Zele, 2014)^29^; pupil constriction in response to light stimuli has been used to evaluate outer retinal rod/cone dysfunction in PD^32^ but intrinsic ipRGC-mediated pupil function has not been investigated. Our primary aim was to perform chromatic pupillometry on optimally medicated PD participants to determine if their ipRGC function is different to people in a healthy control group.

The resting pupil diameter is set by the autonomic nervous system which achieves a dynamic equilibrium between parasympathetic input to the pupillary sphincter and sympathetic input to the dilator muscle^33,34^. The autonomic nervous system is impaired in PD^35,36^ and unmedicated PD patients showed increased pupil diameters after light adaptation, reduced pupil constriction amplitude and a delayed pupil constriction^37^. In a group of mostly (71%, *n* = 12) unmedicated PD patients, the pupillary unrest increased when in darkness^38^. To evaluate the level of autonomic tone in optimally medicated PD patients, the secondary aim was to measure pupillary unrest in the absence of light stimulation.

## Materials and Methods

### Participants

Twenty-nine participants were recruited, comprising of 17 people with PD (mean age = 64.9 years, *SD* = 6.1, 5 female) and 12 control participants (mean age = 59.7 years, *SD* = 4.1, 4 female). PD participants were early stage with a mild to moderate disease severity as assessed by the Unified Parkinson’s Disease Rating Scale^39,40^ (mean score 36.3, *SD* = 12.0) and Hoehn & Yahr^41^ scale (mean score = 1.7, *SD* = 0.6). They were living independently and were cognitively intact (Mini-Mental State Examination^42^ mean score = 29.1, *SD* = 1.1; Addenbrooke’s Cognitive Examination mean score^43,44^ = 91.3, *SD* = 6.9). Participants with PD were optimally medicated during all measurements (mean Levodopa equivalent daily dosage = 597.2 mg, *SD* = 302.1 mg).

A comprehensive ophthalmic examination was completed in all participants. Inclusion criteria included a best corrected visual acuity ≥6/6 (Bailey-Lovie Log MAR Chart), an absence of ocular pathology on slit lamp examination and ophthalmoscopy, and intraocular pressure measured with non-applanation tonometry (iCare, Finland Oy, Helsinki, Finland) within the normal range (<21 mmHg) before dilation and after testing. All participants had normal colour vision as assessed by the Farnsworth D-15. Participants with implanted intra-ocular lenses, medications known to affect pupil size, and other diseases that can affect ipRGC function, including diabetes, were excluded from participation in this study.

Retinal nerve fibre layer (RNFL) thickness was measured using Optical Coherence Tomography (OCT) (Cirrus-HD OCT, Carl Zeiss Meditec, Inc., Dublin, CA, USA and Nidek RS-3000 RetinaScan Advance, Nidek Co., Ltd., Tokyo, Japan). Given the evidence for sleep disturbances in people with PD and that ipRGCs transduce environmental light signals for circadian photoentrainment, sleep quality was assessed in all participants using the Pittsburgh Sleep Quality Index questionnaire (PSQI)^45^.

Experimental protocols were approved by the Queensland University of Technology Human Research Ethics Committee. The Methods were carried out in accordance with the relevant guidelines and regulations, and participants provided informed consent in accordance with the tenets of the Declaration of Helsinki.

### Pupillometer

Light stimuli were generated using a custom built extended Maxwellian-view optical system^31,46–48^. The light from two 5 mm diameter LEDs (short wavelength, ‘blue’ light, λ_max_ = 465 nm; full width half maximum (FWHM) = 19 nm; long wavelength, ‘red’ light, λ_max_ = 638 nm, FWHM = 15 nm) was imaged in the plane of the pupil via two Fresnel lenses (100 mm diameter, 127 mm and 70 mm focal lengths; Edmund Optics, Singapore) and a 5° light shaping diffuser (Physical Optics Corp., California USA) which generated a 35.6° stimulus light. The consensual pupil response was recorded with a Pixelink camera (IEEE-1394, PL-B741 FireWire; 640 × 480 pixels; 60 frames.s^−1^) through a telecentric lens (Computar 2/3″ 55 mm and 2 × Extender C-Mount) under infrared LED illumination (λ_max_ = 851 nm). A chin rest, temple bars and a head restraint maintained alignment in Maxwellian-view. Custom software coded in Matlab (version 7.12.0, Mathworks, Massachusetts USA) controlled stimulus presentation, pupil recording and analysis. Details of the pupillometry measurements are given elsewhere^49,50^.

### Stimuli

**Figure 1:**
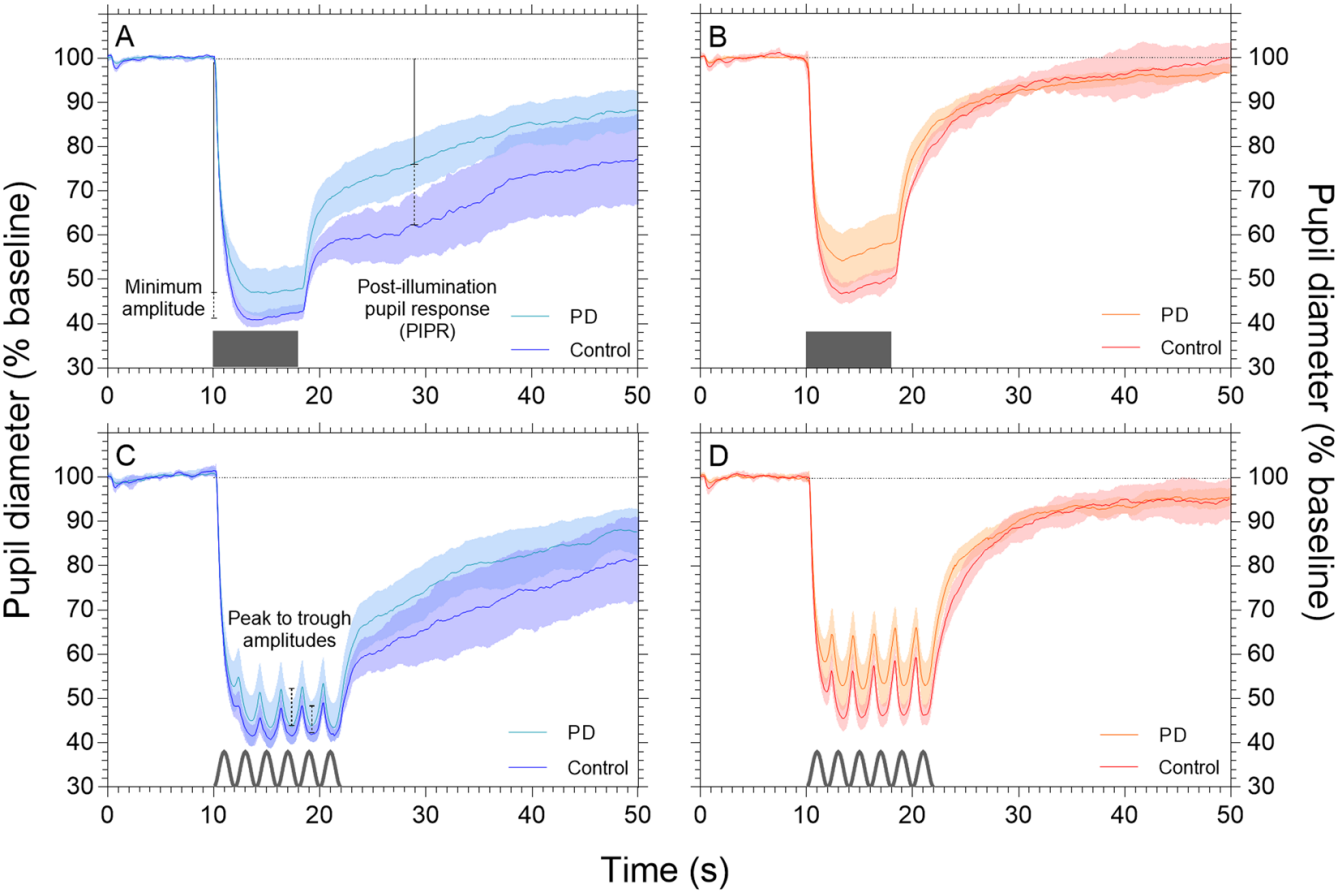
Normalised mean pupil light reflex waveforms (*mean* ± *95% confidence intervals*). Data from the PD group (light tracings, *n* = 17) and the control group (dark tracings, *n* = 12) are shown within each panel, for pulsed (Panels A,B) and sinusoidal (Panels C,D) stimulation in response to both short wavelength (blue tracings, Panels A,C) and long wavelength (red tracings, Panels B,D) light. The pupil metrics are illustrated in Panels A and C (minimum constriction amplitude, PIPR amplitude and peak to trough amplitude) and schematics of the test stimuli are depicted on the abscissa. To control for individual differences in baseline pupil diameter, the data are normalised to the first 10 s of recording.

The light stimulation protocol consisted of a 10 s pre-stimulus baseline recording, pulsed (8 s rectangular) or phasic (12 s, 0.5 Hz sinusoidal) stimulus presentation, and a 40 s post-stimulus recording period (see Fig. 1A,B and C,D for the stimulus waveform). The corneal irradiance of the short and long wavelength stimuli were equated to 15.1 log photons.cm^−2^.s^−1^. Given the older age of the participants, retinal irradiances were estimated using an age-related model of changes in the optical density of the media of the eye (cornea, lens, aqueous and vitreous humours) for stimuli greater than 3° in diameter:^51^ Average short wavelength attenuation was 0.54 log units in the PD group and 0.50 log units in the control group. Average long wavelength attenuation was 0.16 log units for both groups, invariant to group membership and age. To account for the bistability of melanopsin^52^ and participant fatigue^47,53^ stimuli were alternated, beginning with the long wavelength stimulus followed by the short wavelength stimulus. Two recordings for each wavelength of the pulsed and sinusoidal stimulation were obtained and averaged prior to analyses. Pupillary unrest was recorded in the dark for 5 minutes at the end of the pulsed and sinusoidal testing to measure autonomic tone and fatigue (see *Pupil Metrics and Analyses* section). Each participant therefore underwent a total of 9 trials during a recording period lasting approximately 1.5 hours.

### Pupil Metrics and Analyses

Short wavelength light stimulation with high melanopsin excitation activated intrinsic ipRGC inputs to the pupil control pathway^24–26^. Long wavelength stimuli with low melanopsin excitation biased activation to the extrinsic outer retina photoreceptors and was thus a control stimulus with minimal intrinsic ipRGC activation^24,31^.

To investigate the interaction between inner and outer retina photoreceptor inputs to the pupil control pathway during pulsed stimulation, constriction amplitude was measured^28,31^. To determine the interaction between inner and outer retinal contributions to the phasic pupil response of the dark-adapted pupil, two parameters were calculated – the peak to trough amplitude^31^, and the Phase Amplitude Percentage (PAP: (long wavelength peak to trough – short wavelength peak to trough)/long wavelength peak to trough)^29^.

To assess intrinsic melanopsin signalling, the PIPR amplitude can be measured at any time ≥1.7 s after stimulus offset^27^. The melanopsin-mediated PIPR under short wavelength conditions demonstrates a sustained constriction (that is, a reduction from baseline diameter that persists). In contrast, the PIPR amplitude to long wavelength stimulation is less sustained and rapidly returns to baseline due to the lower sensitivity of melanopsin at long wavelengths^6,28^. We calculated the optimal timing of the PIPR metric given our equipment, sample, and stimulus conditions: The control group data for the pulsed and sinusoidal PIPR data were averaged within the short and long wavelength conditions; subtracting the short from long wavelength data determined the timing of the largest difference between these retinal inputs to the PIPR, which was the 1 s window^30^ of the 11th second after light offset. Thus, the PIPR value used for all analyses (both PD and control groups) was 11 s after light offset.

In order to quantify changes in pupillary unrest (hippus, spontaneous oscillations of the pupil primarily driven by central changes in autonomic tone)^54^ that may differ with disease status, we measured pupil diameter in the dark for 5 minutes at the end of the experimental session. The power of pupillary unrest was characterised with the root-mean-square (RMS) of the unrest data. To remove low frequency noise and slow drifts in the pupil data, but to retain higher frequency oscillations (~3 to ~7 Hz) associated with PD tremor^55–57^, a 1 Hz high-pass filter was applied. Dominant frequencies (Hz and dB) of pupillary unrest were then measured using Fast Fourier Transform^58,59^, and disorder in the pupillary unrest was characterized with sample entropy^60^. Low sample entropy values indicate high signal regularity and high sample entropy values indicate low signal regularity. In addition, the average pupillary unrest index (PUI) was calculated for each individual using the method of Lüdtke *et al*. (1998)^61^, over a shortened duration of five minutes to minimise fatigue. The PUI calculates the average pupil diameters at a sample frequency of 1.526 Hz, acting as a low pass filter, and sums their absolute differences. It is thus an additive measure of consecutive pupil diameters that quantifies pupil oscillation variability, and has been used to estimate sleepiness during recording periods of ~11 min^61^.

Each pupil tracing was individually visualised and data due to blinks were linearly interpolated in Matlab. In order to minimise the correlations between the pupil light reflex metrics when expressed in millimetres^62^, the data were normalised to the average pupil diameter of the first 10 seconds and expressed as percentage baseline units. The non-normally distributed data for the PD and control groups were compared using independent samples Mann-Whitney U tests. Correlations within the PD group data were explored using Spearman’s rank order test. All statistical analyses were performed in SPSS Statistics (v23.0, IBM, Armonk, NY, USA) using two-tailed tests with an alpha level of *p* < 0.05.

### Procedure

Participants with PD were assessed for disease severity (UPDRS, H&Y) and cognitive impairment (MMSE) prior to visual testing. All participants were provided the PSQI and instructed in its use (sent via mail and returned on the day of testing), to assess their quality of sleep in the four weeks prior to visual testing. Upon presentation participants had a comprehensive ophthalmic exam, before dilation of their stimulated eye (Tropicamide 0.5% w/v, Bausch & Lomb). Once the pupil had fully dilated the participant was briefed of the protocols and aligned in the pupillometer. All pupillometry was conducted in the dark and before each trial participants adapted to the dim room illumination (<1 lux) for 7 minutes. Between trials the participants were permitted to remove their head from the pupillometer but remained seated. Following pupillometry, participants had their fundus and lens examined (slit lamp), RNFL thickness measured via OCT, and IOP re-assessed. The entire experimental and ophthalmic testing was completed within two hours.

### Data availability

The datasets generated during and/or analysed during the current study are available from the corresponding author on reasonable request.

## Results

The RNFL thickness was similar between the PD group (*median* = 93.00 μm, *interquartile range (IQR)* = 19.50) and control group (*median* = 89.50 μm, *IQR* = 21.00) (*p* = 0.902). Sleep quality was reduced in the PD group (*median* = 7.00, *IQR* = 4.00) compared to controls (*median* = 4.00, *IQR* = 3.00), but this difference was not significant (*p* = 0.264) and groups did not differ along derived 2-factor dimensions of sleep quality (*p* = 0.517) and sleep efficiency (*p* = 0.578)^63^.

**Figure 2:**
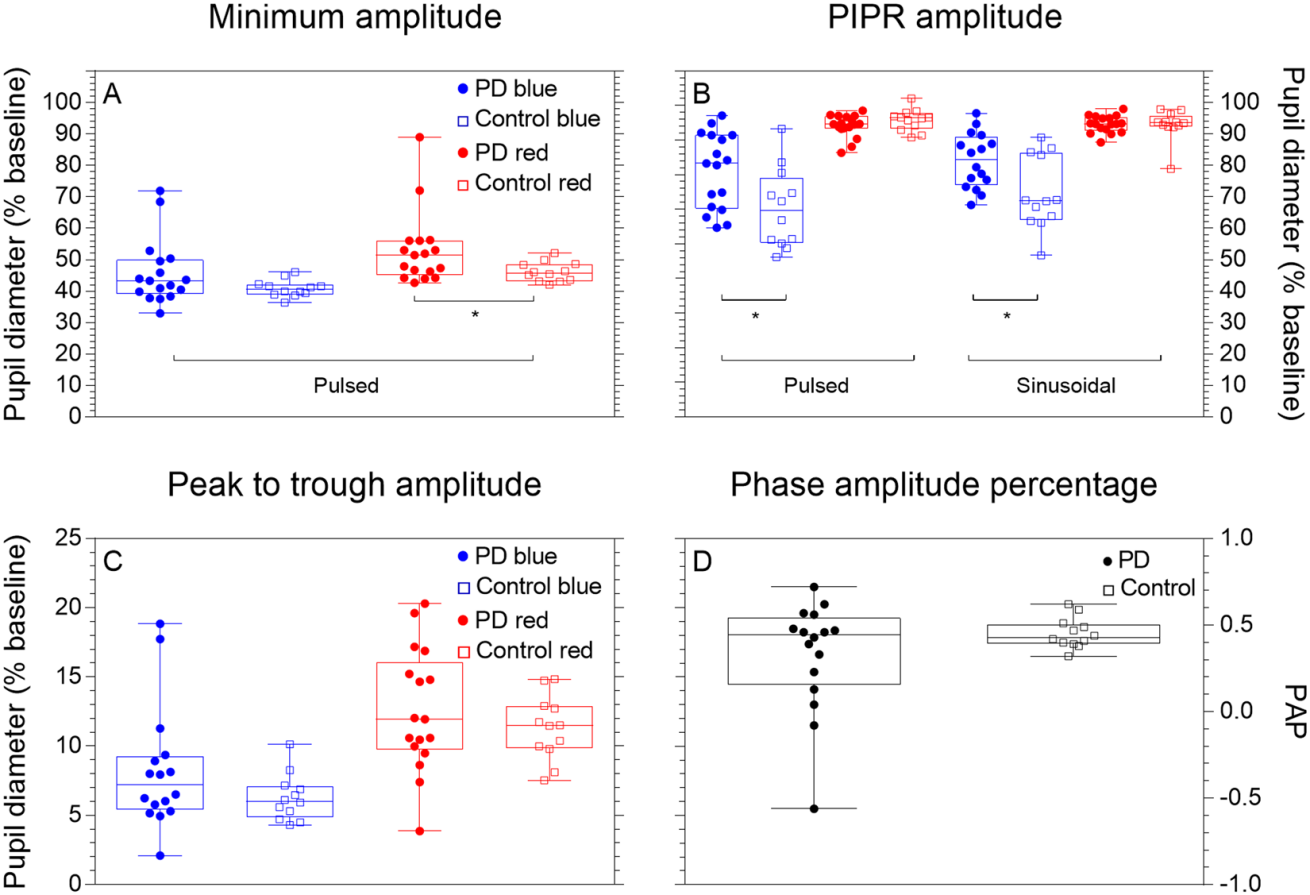
Pupil metrics in response to pulsed and sinusoidal stimulation. The minimum amplitude (Panel A), PIPR amplitude (Panel B), Peak to trough amplitude (Panel C) and phase amplitude percentage (Panel D) are depicted. Data in Panel A are derived from pulsed stimulation and data in Panels C and D are derived from sinusoidal stimulation. Each data point represents an individual’s mean data, boxplots depict the quartiles and whiskers the range. Asterisks indicate a significant difference between groups (*p* < 0.05).

The pupil light reflex for the control and PD groups in response to the pulsed (Fig. 1A,B) and sinusoidal stimuli (Fig. 1C,D) demonstrate reduced PIPR amplitudes (higher % baseline, see also Fig. 2) for short wavelength stimulation.

Box plots (Fig. 2) show all participant data for the minimum amplitude (pulsed stimuli) and PIPR amplitude (pulsed and sinusoidal stimuli). The minimum pupil constriction amplitude for short wavelength pulsed stimulation was similar between the PD (*median* = 43.35%, *IQR* = 10.57%) and control groups (*median* = 39.93%, *IQR* = 2.79%) (*p* = 0.079), whereas the minimum constriction amplitude for long wavelength pulsed stimulation was reduced in the PD group (*median* = 51.48%, *IQR* = 9.73%) compared to the control group (*median* = 46.10%, *IQR* = 6.05%) (*p* = 0.034). The melanopsin-mediated PIPR was measured from the pulsed and sinusoidal pupillometry protocols. For short wavelength stimuli that have a high melanopsin excitation, the pulsed PIPR amplitude was 14.73% higher in PD participants (*median* = 80.32%, *IQR* = 23.16%) compared to controls (*median* = 65.59%, *IQR* = 20.52%) (*p* = 0.018), indicating reduced melanopsin contributions to this process (i.e., closer to baseline diameter in the PD group than controls). Similarly, short wavelength sinusoidal PIPR amplitude was 12.96% higher in the PD group (*median* = 81.72%, *IQR* = 15.21%) compared to controls (*median* = 68.76%, *IQR* = 21.32%; *p* = 0.011). As expected, the long wavelength (with minimal melanopsin excitation) PIPR amplitude was not different between groups for either pulsed (*p* = 0.325) or sinusoidal (*p* = 0.556) stimulation.

To determine if the short wavelength pulsed PIPR amplitude was associated in the PD group with sleep quality (PSQI), clinical symptom severity (UPDRS), RNFL thickness or medication dosage (LEDD), we performed Spearman’s rank-order correlations; no statistically significant correlations were observed (Table 1).

**Table 1:** Spearman’s rank-order correlations between pulsed short wavelength PIPR amplitude and PD markers.

	PIPR	RNFL	UPDRS	LEDD	PSQI
PIPR	1	0.11 (0.68)	0.14 (0.60)	0.24 (0.35)	0.26 (0.32)
RNFL	0.11 (0.68)	1	−0.25 (0.34)	−0.17 (0.51)	0.07 (0.78)
UPDRS	0.14 (0.60)	−0.25 (0.34)	1	−0.11 (0.67)	0.12 (0.65)
LEDD	0.24 (0.35)	−0.17 (0.51)	−0.11 (0.67)	1	0.48 (0.05)
PSQI	0.26 (0.32)	0.07 (0.78)	0.12 (0.65)	0.48 (0.05)	1

*Note:* Data are expressed as *correlation coefficient* (*p value*). PIPR = Post-illumination pupil response, RNFL = Retinal nerve fibre layer thickness, UPDRS = Unified Parkinson’s Disease Rating Scale, LEDD = Levodopa equivalent daily dosage, PSQI = Pittsburgh sleep quality index. *n* = 17.

In response to sinusoidal stimulation, the peak to trough amplitude and the phase amplitude percentage (PAP) of the phasic pupil response (Fig. 2C,D respectively) shows more variability in participants with PD than controls, independent of stimulus wavelength. With short wavelength lights that have high melanopsin excitation (Fig. 2C) the peak to trough amplitude trended to increase in the PD group (*median* = 7.95%, *IQR* = 3.57%), which is indicative of reduced melanopsin contributions compared to controls (*median* = 5.59%, *IQR* = 2.20%), but this difference was not significant (*p* = 0.205). Similarly, under long wavelength stimulation with low melanopsin excitation (Fig. 2C), the peak to trough amplitude did not differ between the PD group (*median* = 12.03%, *IQR* = 6.41%) and controls (*median* = 11.48%, *IQR* = 3.18%) (*p* = 0.471). The median PAP did not significantly different between groups (*p* = 0.537; Fig. 2D).

**Figure 3:**
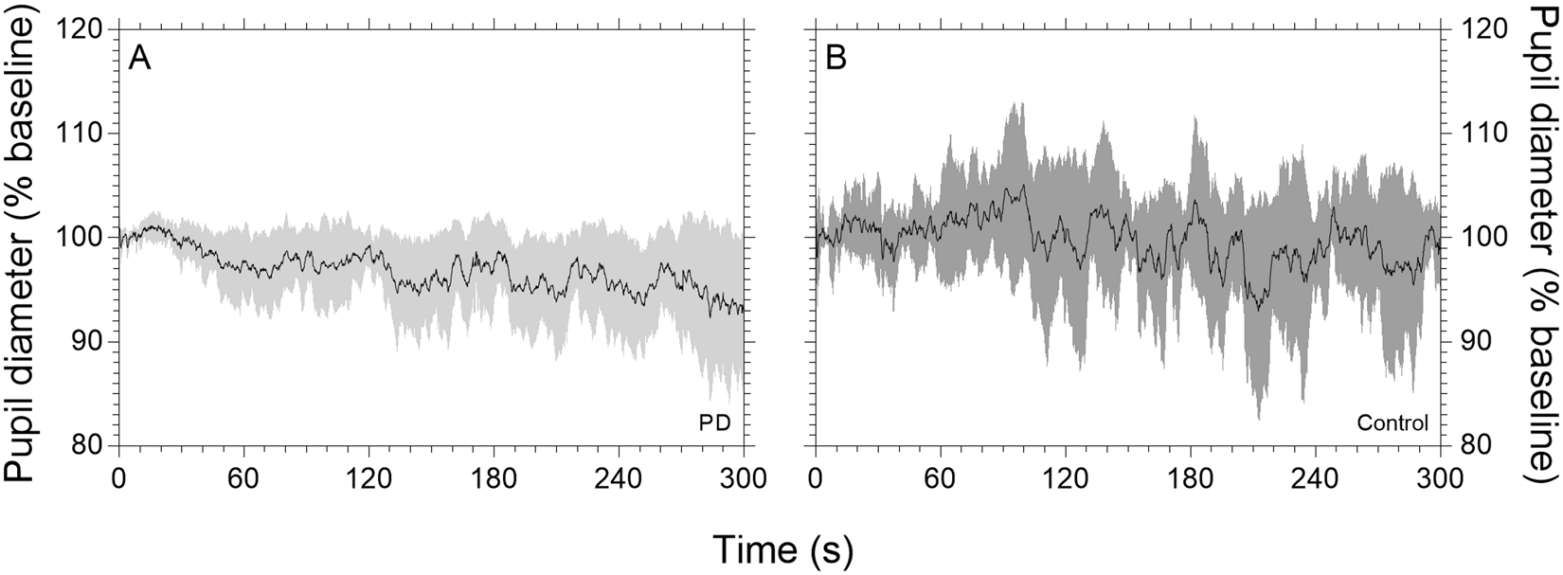
Normalised pupillary unrest in darkness (*mean* ± *95% confidence intervals*). Data from PD group (light tracings, *n* = 17) and control group (dark tracings, *n* = 12) are shown in panels A and B respectively. To control for individual differences in baseline pupil diameter, the data are normalised to the first 10 s of recording.

Pupillary unrest assessed autonomic tone and fatigue, and mean waveforms are shown for the PD and control groups in Fig. 3A,B respectively. Metrics derived from the pupillary unrest recordings are given in Table 2; the PD and control groups did not statistically differ on any metric.

**Table 2:** Medians and interquartile ranges of the pupillary unrest metrics.

	RMS	Dominant frequency (Hz)	Dominant frequency (dB)	Sample entropy	Pupillary unrest index
PD	4.63 (2.20)	1.10 (0.14)	−8.87 (3.92)	0.25 (0.79)	4.69 (2.95)
Control	4.82 (1.48)	1.09 (0.10)	−8.71 (1.79)	0.27 (1.17)	4.88 (6.49)
*p* value	>0.999	0.683	0.507	>0.999	0.683

*Note*: Values are displayed as *Median* (*IQR*).

## Discussion

The melanopsin mediated PIPR to short wavelength stimulation and the pupil constriction amplitude in response to long wavelength stimulation was dysfunctional in optimally medicated individuals with PD. Pupillary unrest however, was not significantly different between the PD and control groups, neither was there a significant sleep deficit as assessed with the PSQI.

The reduction in the PIPR amplitude in the PD group indicates that melanopsin-mediated ipRGC inputs to pupil control pathway are impaired, and that this effect size is both large and clinically relevant (difference between medians = 17.49%). Reduced ipRGC function has been associated with impaired sleep^64,65^ and while there was reduced sleep quality in patients with PD compared to the control group, this difference was not statistically significant. We acknowledge however that alternative methods of sleep assessment such as polysomnography may be more sensitive than the PSQI in detecting sleep deficits. Even so, the observed ipRGCs dysfunction indicates the pathophysiology of circadian and sleep disorders in PD patients includes a retinal source that leads to aberrant signalling to circadian centres.

The PIPR amplitude was reduced in response to both pulsed and sinusoidal stimulation in the PD group, and these deficits were observed in the PD participants with no retinal thinning as compared to controls. Previous studies have identified reduced RNFL thickness in people with PD including at the early- to mid-stage^66,67^. That the PD group did not statistically differ in RNFL thickness compared to controls is consistent with the early stage diagnosis based upon their clinical UPDRS and H&Y scores^68^. Because ipRGCs have low redundancy compared to canonical retinal ganglion cells^5,7^, functional ipRGC deficits may be measureable before a reduction in ganglion cell numbers is detected using conventional ophthalmic imaging.

Given the aetiology of PD, deficits in ipRGC function could be linked to a reduction in dopamine expression. IpRGCs form retinal circuits with dopaminergic amacrine cells and may themselves be sensitive to DA through feedback loops^22,69–71^. The PIPR amplitude is reduced in patients with type II diabetes without diabetic retinopathy^53^, which in rodent models features decreased retinal dopamine^72,73^. Post-mortem examination reveals that DA cell morphology is abnormal in the PD retina, with reductions in both DA and DA’s synthesising enzyme tyrosine hydroxylase^74,75^, although retinal DA is reduced in unmedicated but not medicated patients with PD in one study^76^. Alternate hypotheses include deficiencies in the cholinergic inputs to the pupil control system^77^, compatible with cholinergic gait disturbances in PD^78,79^; or reduced ipRGC signaling due to α-synuclein deposition within the inner plexiform and ganglion cell layers^80,81^.

The pupil constriction response to long wavelength light is unaffected by yellowing of the lens with ageing and represents extrinsic photoreceptor contributions to the ipRGCs. With a small (5.38%) but statistically significant difference, this pathway is impaired in the PD group. Consistent with this observation, but in unmedicated PD with a light-adapted paradigm (1200 Lux for 10 minutes), slower pupil constriction latency and timing as well as a larger (12.58%) reduction in constriction amplitude has been observed^37^. A suboptimal dark adaptation state^82^, linked to abnormal dopamine expression in the PD retina, may underpin such dysfunction. Pupillometric deficits in outer retinal-mediated responses may parallel visual performance deficits in the central and peripheral retina of PD patients, including colour vision, contrast sensitivity, and electroretinography (for review see Bodis-Wollner)^83^.

Pupillary unrest metrics did not differ between the PD and control groups, exhibiting both low entropy, indicating signal regularity, and similar dominant frequencies. In contrast, Jain *et al*.^38^ reported increased pupillary unrest during a longer 11-minute protocol in a predominantly unmedicated PD group (71%) of similar disease severity to our sample (H&Y = 1.7 (0.6), UPDRS = 20.5 (9.6)). Medication may therefore influence the resting pupil size, obscuring deficits in pupillary unrest mediated by the autonomic system, whereas the light-dependent PIPR amplitude is dysfunctional in optimally medicated populations.

This initial assessment of melanopsin-mediated ipRGC function in people with PD demonstrates that the PIPR, a marker of melanopsin pathway function, is disrupted in optimally medicated individuals with PD. Given that the PIPR amplitude is uncorrelated with both clinical ratings of the disease and medication dosage (Table 1), further studies should assess the potential to detect prodromal PD. Longitudinal studies testing the hypothesis that ipRGC dysfunction increases with disease duration should explore the links between the retina and circadian disorders using more sensitive measures of circadian function. On the basis that ipRGCs are the primary conduit for entrainment to the solar day^15^ and innervate brain centres involved in sleep/wake regulation^11^, ipRGC dysfunction may play an important role in the pathophysiology of sleep and circadian rhythms in PD.
